# Silicon supplementation enhances productivity, water use efficiency and salinity tolerance in maize

**DOI:** 10.3389/fpls.2022.953451

**Published:** 2022-11-23

**Authors:** Abdullah H. Alayafi, Samir G. M. Al-Solaimani, Mohamed H. Abd El-Wahed, Fahad M. Alghabari, Ayman El Sabagh

**Affiliations:** ^1^ Department of Arid Land Agriculture, Faculty of Meteorology, Environment & Arid Land Agriculture, King Abdulaziz University, Jeddah, Saudi Arabia; ^2^ Agronomy Department, Faculty of Agriculture, Kafrelsheikh University, Kafrelsheikh, Egypt; ^3^ Department of Field Crops, Faculty of Agriculture, Siirt University, Siirt, Turkey

**Keywords:** water salinity, silicon, trickle irrigation system, tolerance, *Zea mays* L.

## Abstract

Drought and salinity stress severely inhibits the growth and productivity of crop plants by limiting their physiological processes. Silicon (Si) supplementation is considerd as one of the promising approaches to alleviate abiotic stresses such as drought and salinity. In the present study, a field experiment was conducted over two successive growth seasons (2019-20) to investigate the effect of foliar application of Si at two concentrations (1 and 2 kg Si ha^-1^) on the growth, yield and physiological parameters of three maize cultivars (ES81, ES83, and ES90) under three levels of irrigation salinity) [1000 (WS_1_), 2000 (WS_2_) and 3000 (WS_3_) mg L^-1^NaCl]. In this study, A trickle irrigation system was used. Si application significantly mitigated the harsh effects of salinity on growth and yield components of maize, which increased at all concentrations of Si. In irrigation with S3 salinity treatment, grain yield was decreased by 32.53%, however, this reduction was alleviated (36.19%) with the exogenous foliar application of Si at 2 kg Si ha^-1^. At salinity levels, Si application significantly increased maize grain yield (t ha^-1^) to its maximum level under WS of 1000 mg L^-1^, and its minimum level (Add value) under WS of 3000 mg L^-1^. Accordingly, the highest grain yield increased under Si application of 2 kg Si ha^-1^, regardless of salinity level and the cultivar ES81 achieved the highest level of tolerance against water salinity treatments. In conclusion, Application of Si at 2 kg Si ha^-1^ as foliar treatment worked best as a supplement for alleviating the adverse impacts of irrigation water salinity on the growth, physiological and yield parameters of maize.

## Introduction

The main challenge for modern agriculture is to meet the nutritional needs of the world’s growing population in an economically and environmentally sustainable manner. Food production is required to increase by 70% to satisfy the growing population demand by the year 2050 (Godfray et al., 2010). Among the most cultivated food crops worldwide, maize (*Zea mays* L.) ranks third after wheat and rice (Malcovska et al., 2014). It is a staple and primary food for most people in developing countries, while it is used as animal feed and other byproducts in developed countries. Due to its higher-yielding nature, maize is a key crop for densely populated countries to ensure food security. Several countries have also made it one of their most important forage and cereal crops (FAO, 2017). Maize production needs to be increased worldwide for many purposes including human nutrition, fodder, pharmaceuticals, and industrial manufacture (Ayyar et al., 2019). In addition to being used as a raw material for antibiotics, it is used in the manufacturing of starch, ethanol, and plastics (Abd El-Wahed and Ali, 2013). However, to meet the increasing demand for animal and human consumption in many ways, studies have suggested that maize production must double especially in developing countries. The global food demand is increasing due to expanding population and their subsequent consumption, and it will be a great challenge for food security under climate change and changes in land-use scenarios (Islam et al., 2022), which comes with direct and indirect adverse effects on the form of abiotic stresses on crops (Shahzad et al., 2019; Ullah et al., 2020; Hamid et al., 2021; Naveed et al., 2021). The production of maize in many arid and semi-arid regions, including Saudi Arabia, is sverely affected by abiotic stress factors (Ahmad et al., 2019; Nazir et al., 2021; Thind et al., 2021).

Salinity stress is one of the most detrimental abiotic stresses severely affecting the agricultural productivity and threatening global food security (Daliakopoulos et al., 2016; Niamat et al., 2019; Islam et al., 2022). Salinity affects approximately 62 million hectares (20%) of the agricultural area around the world (Yoon et al., 2009). More than half of irrigated land is predicted to be salt affected by 2050 (Hamayun et al., 2010). Maize is a moderately sensitive crop to salinity stress (Carpici et al., 2010). Generally, salinity has an impact on seed germination, plant growth, and development, as well as reducing osmotic potential, increasing ionic toxicity, and disrupting water balance, nutrient absorption, and hampering the biochemical and microbial activities which severely reduce the productivity of various crops (Thorne et al., 2020; Askari-Khorasgani et al., 2021; Yasir et al., 2021). Higher NaCl concentration decreased fresh and dry biomass, and relative growth rate, as well as leaf area ratio in two *Zea mays* cultivars (salt sensitive Trihybrid 321 and salt tolerant Giza 2) Mansour et al. (2005). Hence, to reduce the future impact of salt-induced challenges, an environment friendly management strategy must be implemented. In this regard, application of silicon (Si) has emerged as an emerging and promising option to mitigate salt stress (Dhiman et al., 2021).

Si is the second most abundant element in the earth’s crust after oxygen, and it is mainly present in the form of SiO_2_ in soil (Sommer et al., 2006). Plants can absorb Si in the form of silicic acid [Si(OH)], which is often limiting in the soil (Coêteí-Beaulieu et al., 2009). It has been reported that soils contain 100-500 μmol L^-1^ silicic acid, although its exact availability varies depending on soil type, temperature, and pH (Sommer et al., 2006). Si applied to the plants alleviates drought and salinity stress (Olivera et al., 2019). It has been documented that Si can promote maize growth under saline conditions (Sattar et al., 2016). According to Zhu and Gong (2014), the mechanisms underlying the mitigation of Si-mediated salt stress include: (a) maintaining optimal water content; (b) enhancement of photosynthesis and curbing the rate of transpiration; (c) Reducing oxidative stress by mitigating ion toxicity and (d) biosynthetic regulation of solutes and plant hormones. In this context, Al-aghabary et al. (2005) observed the increased activities of antioxidant enzymes with significant photochemical efficiency of photosystem II (PSII) with the application of Si under salt stress. Si fertilization might be a quick and economical method for improving crop yields under salt stress as compared to any other method for implementation of small-holder farmers. In this study, we evaluate the effects of foliar application of Si on maize growth, development, and water use efficiency (WUE) in response to salinity stress, and determined the optimum level of exogenous Si application to mitigate water salinity for maize production in pedoclimatic conditions of arid regions.

## Materials and methods

### Study area

**Figure 1:**
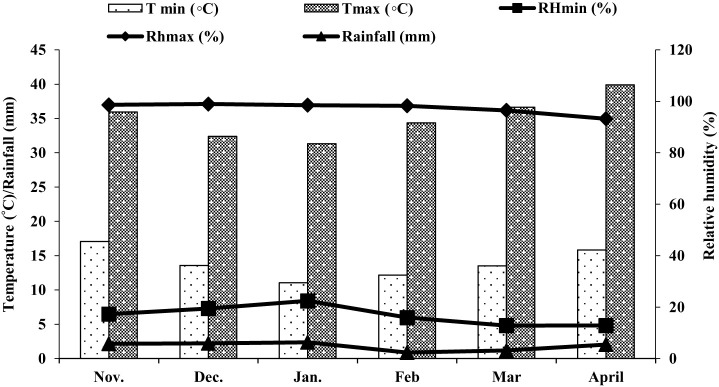
The average meteorological data of the field during the experimental period.

Field trials were conducted at the Agricultural Research Station, Hada Al-Sham (21°48’ 3” N, 39°43’25”E), Jeddah, Saudi Arabia during 2019-20 and 2020-21. Weather data on the monthly average temperature, relative humidity, and rainfall in the field during the experimental period were recorded regularly. During the growing period, the temperature fluctuated from 11.05 to 39.89°C. The average temperature was around 24.4775°C. The minimum humidity of those days was 12.83% and the maximum was 98.96%. similarly, the maximum rainfall was 6.33 mm and the minimum was 2.35 mm. Data are presented in **Figure 1**.

### Soil analysis

Before the start of the experiment, composite soil samples (0-15 cm) were taken from the experimental site and analyzed regarding physicochemical properties (**Table 1**).

**Table 1:** Physical and chemical properties of the experimental soil.

Soil characteristics	Values
pH	7.72
EC (dS m^-1^)	3.35
Sand	84.5
Silt	12.3
Clay	3.2
Soil texture	Loamy Sand
Organic matter (%)	0.5
N (%)	0.03
P (%)	0.001
K (%)	0.06
Ca (%)	0.34
Na (%)	0.04

### Field experiment

Field experiments were laid out in a randomized complete block design with a split-split plot arrangement in triplicates, with saline irrigation as the main plot (WS_1_ = 1000, WS_2_ = 2000, and WS_3_ = 3000 mg L^-1^ NaCl), and subplots contained three cultivars (ES 81, ES 83 and ES90) of maize and foliar application of Si as CaSiO_4_ (0, 1, and 2 kg Si ha^-1^) as the sub-sub plots. **Table 2** shows the characteristics of the three different cultivars of maize.

**Table 2:** The type and strain of maize cultivars used in the experiment.

Cultivars*	Abbreviation	Type	Color	Breeding
Egaseed 81	ES 81	Single hybrid	White	S 2650
				S 758
Egaseed 83	ES 83	Single hybrid	White	S 5146
				S 1053
Egaseed 90	ES 90	Single hybrid	White	S 2823
				S 1053

*Source: Egyptian Agricultural for Seed Production, EGAS.

7The sub-sub plot size was 6 m^2^ (2 ×3 m) with a row to row distance of 50 cm and hill spacing of 30 cm with one plant/hill. At the start of the experiment, chemical fertilization (NPK) was practiced at the recommended rate for corn production in this area. NPK fertilizer (20-20-20) at a rate of 500 kg ha^-1^ was appied in five splits the first dose was applied 15 days after sowing. The second, third, fourth, and fifth doses were administered after 15, 30, 60, and 90 days following the first dose, respectively. Three doses of Si were sprayed at 15, 45, and 75 days after germination.

### Irrigation system

The experimental area was plowed twice, leveled, and then irrigated by a drip irrigation system which contained three plastic tanks (6000 L capacity each), a disk filter, pump, controller, and solenoid valve to control flow time. Each lateral had a diameter of 16 mm and emitters were placed 30 cm apart. Each emitter had a flow rate of 4 L h^-1^ at a pressure of 1bar. The laterals were spaced at 50 cm. Plots involving WS treatments were isolated with 2 m fallow land to avoid the lateral movement of water from one plot to another. Subplots within each WS treatment were isolated by a distance of 0.5 m of fallow land. The water source from the installed container was always full of water *via* the main irrigation network of the farm. The required irrigation water was calculated based on maize crop water requirements.

### Application of irrigation water

Maize plants were irrigated at 2 days intervals by applying the amount of irrigation water required. The daily ET_o_ was computed according to Eq. (1) (Allen et al., 1998) as follows:


(1) $$ETo=\frac{0.408\;\;\Delta (R_{n}-G)+\gamma \frac{900}{T_{mean}+273}u_{2}(e_{s}-e_{a})}{\Delta +\gamma (1+0.34\;u_{2})}$$

Where ETo: is the reference evapotranspiration (mm day^−1^), Δ the slope of the saturation vapor pressure curve at air temperature (kPa C^−1^), Rn the net radiation at the crop surface (MJm^−2^ d^−1^), G Soil heat flux density (MJm^−2^ d^−1^), γ psychometric constant = (0.665×10−3 ×P), kPa C^−1^ (Allen et al., 1998), P is the atmospheric pressure (kPa), U_2_ wind speed at 2 m height (m s^−1^), e_s_ is the saturation vapor pressure (kPa), e_a_ actual vapor pressure (kPa) (e_s_ −e_a_) is the saturation vapor pressure deficit (kPa), and T_mean_ mean daily air temperature at 2 m height (°C). The average of daily ETo was 7.85, 6.31, 6.65, 8.69, 10.73 and 12.83 mm day^−1^ in November, December, January, February, March and April, respectively.

Based on reference evapotranspiration and crop coefficient, crop evapotranspiration of maize was calculated according to Eq. (2):


(2) ETc=ETo×Kc

Where ETc is the crop water requirement (mm. day^−1^) and Kc is the crop coefficient. The lengths of the different crop growth stages were 25, 40, 45, and 30 days for initial stage, crop development stage, mid-season stage and late season stage, respectively and the crop coefficients (Kc) of initial, mid and end stage were 0.70, 1.20 and 0.35, respectively, according to Allen et al. (1998).

The amount of irrigation water applied for each treatment during the irrigation regime was determined by using Eq. (3):


(3) $$IWA=\frac{A\times ETc\times Ii\times Kr}{Ea\times 1000\times (1-LR}$$

Where IWA is the irrigation water applied (m^3^), A is the plot area (m^2^), ETc is the crop water requirements (mm. day^−1^), Ii is the irrigation intervals (day), Ea is the application efficiency (%) (Ea = 85), Kr covering factor and LR is the leaching requirements.

The amount of irrigation water applied was 7764 and 7700 m^3^ ha^-1^ for the first and second seasons, respectively. Irrigation treatments were started after full emergence at which each treatment was irrigated according to prescribed irrigation salinity treatments.

### Evaluation of agronomic traits

At harvesting, 10 plants were randomly chosen from each experimental unit to determine cob fresh and dry weight (g), total fresh and dry weight (g), 100-kernel weight (g), grain yield (t ha^-1^), cob dry weight (t ha^-1^), stover yield (t ha^-1^), harvest index and shelling percentage.

The harvest index (HI) for each treatment was calculated by using Eq. (4):


(4) $$\text{HI}=\frac{\text{Grain yield }\left(\text{t}/\text{ha}\right)}{\text{Total biomass yield }\left(\text{t}/\text{ha}\right)}$$

The shelling percentage for each treatment was calculated by using the Eq. (5):


(5) $$\text{Shelling \%}=\frac{\text{Grain weight }\left(10\text{ cob}\right)\;}{\text{Total weight }\left(10\text{ cobs}\right)}\times 100$$

### Measurement of grain and other biological yields

Data on biological yield were recorded by harvesting three central rows in each plot, the material was sun-dried for several days and weighed, and then converted into biological yield (kg ha^-1^). The ears of the three central rows were separated from the harvested material for the biological yield. The ears were threshed, cleaned and weighed, and then converted into grain yield (kg ha^-1^).

### Water use efficiency

The water use efficiency of the maize was calculated by using Eq. (6) according to Jensen (1980):


(6) $$\text{WUE}=\frac{\text{Seed yield }\left({\text{kg ha}}^{-1}\right)\;}{\text{Irrigation water applied }\left({\text{m}}^{3}{\text{ha}}^{-1}\right)}$$

### Data analysis

All the data associated with physiological indices and agronomical yield were statistically analyzed using analysis of the variance (ANOVA). ANOVA of the treatment means was conducted using the SAS program (SAS Institute, 2006). The statistical comparison of the treatment means was tested by LSD at (*p* ≤ 0.05) according to Steel (1997). All measurements were carried out using three independent biological replicates.

## Results

### Plant fresh and dry weight

Results in **Table 3** showed a significant decrease in cob fresh weight up to 28.36 and 29.82%, in cob dry weight up to 27.54 and 29.13%, in total fresh weight up to 23.11 and 24.56%, and total dry weight up to 22.86 and 24.51% during 1^st^ season and 2^nd^ season, respectively under an increase in WS from 1000 to 3000 mg L^-1^. On the other hand, the results indicated an increase in means of all these parameters with an increase of Si from 0 to 2 kg ha^-1^ up to 20.68 and 22.07% in cob fresh weight, 22.22 and 23.64% in cob dry weight, and 20.11 and 21.39% in total fresh weight, and 19.70 and 21.11% in total dry weight during the two seasons, respectively.

**Table 3:** Fresh and dry weight cob and plant of maize cultivars under salt and Si application during 2019-20 and 2020-21 seasons.

Treatments	Total dry weight (g)		Total fresh weight (g)		Cob dry weight (g)		Cob fresh weight (g)	
	2019-20	2020-21	2019-20	2020-21	2019-20	2020-21	2019-20	2020-21
**Salinity (mg L^-1^)**								
1000	301.04 a	286.16 a	733.50 a	689.64 a	114.33 a	105.00 a	227.15 a	210.98 a
2000	262.82 b	246.84 b	641.94 b	596.83 b	97.63 b	88.83 b	193.83 b	178.37 b
3000	232.23 c	216.02 c	563.96 c	520.26 c	82.84 c	74.41 c	162.72 c	148.06 c
LSD	18.64	18.13	48.11	44.82	13.4	12.37	26.42	24.60
**Maize cultivars**								
ES 81	261.74 a	257.17 a	648.73 a	616.18 a	102.33 a	94.20 a	201.19 a	187.10 a
ES 83	274.67 a	251.01 ab	656.68 a	604.05 a	98.73 a	90.49 a	196.26 a	180.60 a
ES90	259.69 a	240.84 b	633.99 a	586.50 a	93.75 a	83.55 a	186.26 a	169.70 a
**Silicon application (kg ha^-1^)**								
0	241.38 c	225.33 c	587.40 c	543.36 c	88.16 c	79.58 c	176.08 c	160.87 c
1	265.80 b	250.79 b	646.48 b	603.79 b	98.89 b	90.27 b	195.12 b	180.15 b
2	288.91 a	272.89 a	705.52 a	659.57 a	107.75 a	98.39 a	212.50 a	196.38 a
LSD	12.54	11.21	31.37	29.37	6.97	6.36	13.78	12.64

Values in the column with identical letter(s) do not substantially differ at the 5% level of probability.

### Interaction between silicon and maize cultivars on the plant fresh and dry weight

**Figure 2:**
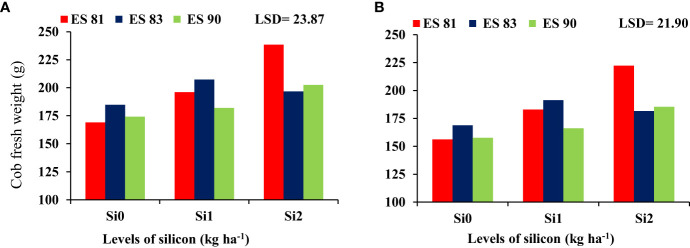
Effect of silicon application on the cob fresh weight of maize cultivars during 2019-20 **(A)** and 2020-21 **(B)**.

**Figure 3:**
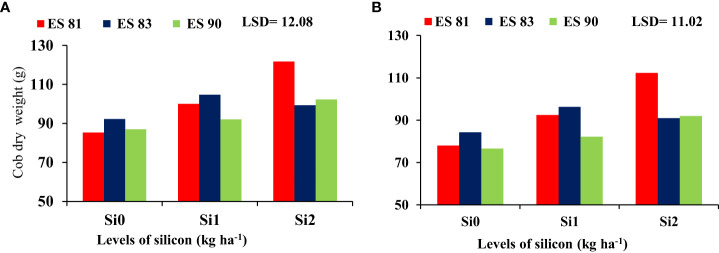
Effect of silicon on the cob dry weight of maize cultivars during 2019-20 **(A)** and 2020-21 **(B)**.

The interaction effect between Si and different maize cultivars indicated significant differences in the fresh and dry weight of cobs, but it was noted no significant difference in the total fresh and dry weight. As shown in **Figure 2**, spraying Si on the maize leaves at rates of 0, 1, and 2 kg ha^-1^ reduced the adverse effects of salinity on the cob fresh weight of maize cultivars under all concentrations of salt levels. Cultivar ES83 gave the highest cob fresh weight under Si 1 kg ha^-1^ concentration compared to the two other maize cultivars, but the cultivar ES81 exceeded in cob fresh weight under 2 kg ha^-1^ Si concentration. Cultivar ES81 resulted in the highest cob dry weight under Si 2 kg ha^-1^ concentration compared to the two other cultivars, while cultivar ES83 exceeded in cob dry weight under 1 kg ha^-1^ Si concentration (**Figure 3**).

**Figure 4:**
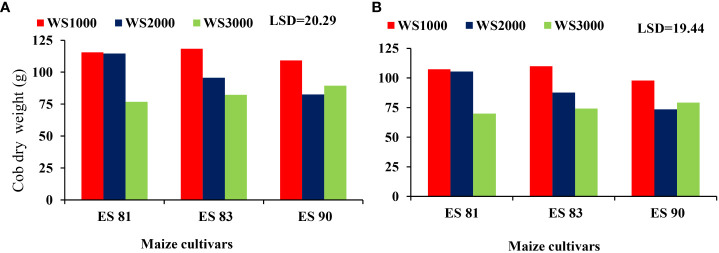
Effect of salinity on the cob dry weight of maize cultivars during 2019/2020 **(A)** and 2020/2021 **(B)**.

The results indicated there was non-significant (*p* ≤ 0.05) effect of WS and maize cultivars on plant fresh and dry weight of the different components (cob fresh weight, total fresh and dry weight) at all salinity levels of 1000, 2000, and 3000 mg L^-1^. However, the ES81 cultivar showed a statistically significant difference (*p* ≤ 0.05), as it achieved the highest mean dry weight at all WS levels compared to the other two cultivars (**Figure 4**).

### Yield and its components

Maize grain yield (t ha^-1^), cob dry weight (t ha^-1^), stover yield (t ha^-1^), and total biomass yield (t ha^-1^) were significantly decreased with WS levels and reduction increased with an increase in salinity level from 1000 to 3000 mg L^-1^ up to 32.53 and 31.28% for grain yield, in cob dry weight up to 42.97 and 42.80%, stover yield up to 23.81 and 23.85%, and in total biomass yield up to 31.78 and 31.62% during 1^st^ and 2^nd^ season, respectively (**Table 4**). On the other hand, the results indicated a significant increase in grain yield up to 36.19 and 36.01%, in cob dry weight up to 27.12 and 27.05%, in stover yield up to 17.28 and 17.33%, and in total biomass yield up to 21.07 and 20.92% during 1^st^ and 2^nd^ season, respectively under an increase in Si concentration from 0 to 2 kg ha^-1^ (Table 4), with no significant differences among the maize cultivars in mean values of these parameters were recorded.

**Table 4:** Effect of silicon on the yield and its components and water use efficiency of the maize cultivars under irrigation water salinity during 2019-20 and 2020-21 seasons.

Treatments	Total biomass (t ha^-1^)		Stover yield (t ha^-1^)		Cob dry weight (t ha^-1^)		Grain yield (t ha^-1^)	
	2019-20	2020-21	2019-20	2020-21	2019-20	2020-21	2019-20	2020-21
**Salinity (mg L^-1^)**								
1000	26.40 a	25.11 a	15.37 a	14.76 a	11.03 a	10.35 a	8.36 a	8.47 a
2000	22.35 b	21.27 b	13.60 b	13.06 b	8.74 b	8.21 b	7.23 b	7.43 b
3000	18.01 c	17.17 c	11.71 c	11.24 c	6.29 c	5.92 c	5.64 c	5.82 c
LSD	1.70	1.60	1.14	1.09	1.657	1.538	0.405	0.415
**Maize cultivars**								
ES 81	22.45 a	21.37 a	13.77 a	13.22 a	8.67 a	8.14 a	7.16 a	7.33 a
ES 83	22.12 a	21.05 a	13.33 a	12.80 a	8.79 a	8.25 a	6.97 a	7.14 a
ES90	22.20 a	21.13 a	13.59 a	13.04 a	8.60 a	8.08 a	7.09 a	7.26 a
**Silicon application (kg ha^-1^)**								
0	20.07 c	19.12 c	12.44 c	11.94 c	7.63 c	7.17 c	5.83 c	5.97 c
1	22.39 b	21.31 b	13.66 b	13.11 b	8.73 b	8.20 b	7.46 b	7.64 b
2	24.30 a	23.12 a	14.59 a	14.01 a	9.70 a	9.11 a	7.94 a	8.12 a
LSD	0.948	0.903	0.675	0.648	0.478	0.445	0.145	0.149

Values in the column with identical letter(s) do not substantially differ at the 5% level of probability.

### Interaction between irrigation water, salinity, and different maize cultivars on yield and its components

**Figure 5:**
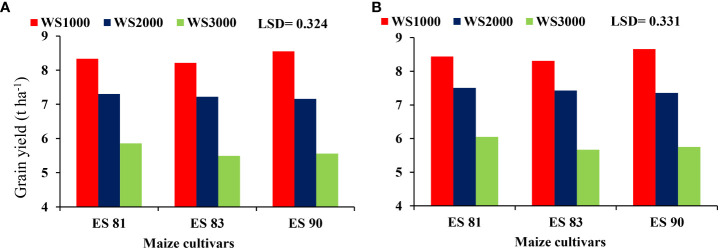
Effect of salinity on the grain yield of maize cultivars during 2019-20 **(A)** and 2020-21 **(B)**.

The interaction results between the WS and maize cultivars had a major effect on the grain yield but not on cob dry weight, stover yield, and total biomass yield in both seasons. The reduction in values of the parameters means an increase with an increase in salinity level to reach its lowest values at 3000 mg L^-1^ in all cultivars. The interaction effect of WS and maize cultivars ES81, ES83, and ES90 on the grain yield shows that the three cultivars attained the highest grain yield under 1000 mg L^-1^ salinity level and their minimum grain yield at salinity level 3000 mg L^-1^ (**Figure 5**). Cultivar ES81 resulted in the highest grain yield than other cultivars under salt stress.

### Interaction between irrigation water salinity and silicon on yield and its components

Mean values of maize grain yield (t ha^-1^), cob dry weight (t ha^-1^), stover yield (t ha^-1^), and total biomass yield (t ha^-1^) were significantly decreased with an increase in WS levels. But Si foliar application at the rate from 0 to 2 kg ha^-1^ significantly increased and improved the mean values of these parameters under all salinity levels to 40.18, 33.33, and 24.10% during 1^st^ season, and 40.67, 38.66, and 27.23% during 2^nd^ season for the grain yield; 37.80, 15.81, and 15.91% during 1^st^ season, 34.44, 25.58, and 25.43% during 2^nd^ season for cob dry weight; 10.01, 17.86, and 16.66% during 1^st^ season, and 9.93, 27.29, and 27.31% during 2^nd^ season for the stover yield; 20.75, 17.07, and 17.10% during 1^st^ season, and 20.52, 26.60, and 20.00% during 2^nd^ season for the total biomass yield under salinity level 1000, 2000 and 3000 mg L^-1^, respectively.

**Figure 6:**
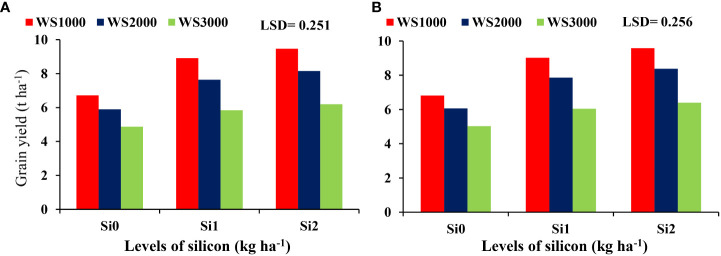
Effect silicon on the grain yield under salinity during 2019-20 **(A)** and 2020-21 **(B)**.

The mean values of these parameters were increasing with an increase in Si concentration under all salinity levels. Si application at all concentration levels of 0, 1, and 2 kg ha^-1^ increased the maize grain yield to the maximum level under salinity level 1000 mg L^-1^, and the minimum increases were under salinity level 3000 mg L^-1^. Under all salinity levels, the highest grain yield was under the Si concentration of 2 kg ha^-1^ (**Figure 6**).

**Figure 7:**
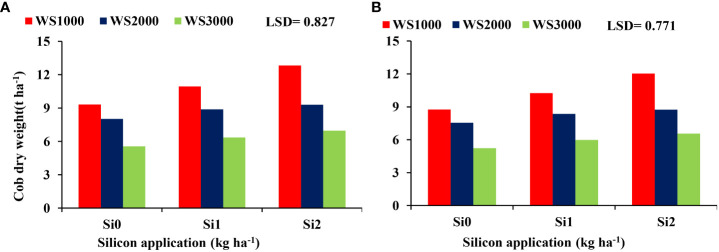
Effect of silicon on the cob dry weight under salinity 2019-20 **(A)** and 2020-21 **(B)**.

The interaction between salinity and Si effect on the cob dry weight indicated that the improvement of cob dry weight due to Si under salinity stresses was most prominent at salinity level 1000 mg L^-1^ and the least at 3000 mg L^-1^. The most effective Si concentration was 2 kg ha^-1^ resulting in the highest cob dry weight under all salinity levels (**Figure 7**).

### Regulation of maize yield components in response to salinity

The 100 kernel weight, shelling percentage, and WUE were significantly decreased by the WS levels, and the reduction gradually increased with an increase in salinity levels from 1000 to 3000 mg L^-1^ up to 38.54 and 38.57% for 100 kernel weight, 7.90 and 7.96% for shelling percentage and 32.71 and 31.81% for WUE during 2019-20 and 2020-21, respectively. However, there was no significant difference in the harvest index (HI) during 2019-20 and 2020-21 (**Table 5**). Conversely, the results indicated a significant increase in 100 kernel weight up to 32.58 and 32.59%, HI up to 10.34 and 12.90%, shelling percentage up to 7.90 and 7.96%, and WUE up to 36.00 and 36.36% during 1^st^ and 2^nd^ seasons, respectively due to increase of Si concentration from 0 to 2 kg ha^-1^, and with no significant differences were recorded among the maize cultivars in mean values of these parameters.

**Table 5:** Number of 100 kernel weight, harvest index, shelling percentage, and water use efficiency under the effects of irrigation water salinity, maize cultivars, and silicon application during the 2019-20 and 2020-21 seasons.

Treatments	Water use efficiency (kg m)^-3^		Shelling percentage		Harvest index (%)		100 kernel weight (g)	
	Season 1	Season 2	Season 1	Season 2	Season 1	Season 2	Season 1	Season 2
**Salinity (mg L^-1^)**								
1000	1.07 a	1.10 a	77.00 c	83.06 c	0.31a	0.35a	34.85a	33.11a
2000	0.93 b	0.96 b	83.17 b	91.06 b	0.32a	0.35a	28.06b	26.66b
3000	0.72 c	0.75 c	89.69 a	98.47 a	0.31a	0.34a	21.42c	20.34c
LSD	0.051	0.055	11.06	11.85	–	–	5.02	4.76
**Maize cultivars**								
ES 81	0.92 a	0.94 a	83.94a	91.55a	0.31a	0.34a	28.03a	26.63a
ES 83	0.89 a	0.92 a	81.10a	88.52a	0.31a	0.34a	29.41a	27.94a
ES90	0.91 a	0.93 a	84.82a	92.51a	0.31a	0.34a	26.89a	25.54a
**Silicon application (kg ha^-1^)**								
0	0.75 c	0.77 c	78.10c	85.19c	0.29b	0.31b	24.03c	22.83c
1	0.96 b	0.99 b	87.48a	95.42a	0.33a	0.65a	28.44b	27.01b
2	1.02 a	1.05 a	84.27b	91.97b	0.32a	0.35	31.86a	30.27a
LSD	0.018	0.019	4.15	4.50	0.01	0.016	2.49	2.37

Values in the column with identical letter(s) do not substantially differ at the 5% level of probability.

### Interaction among irrigation, water salinity, and silicon

**Figure 8:**
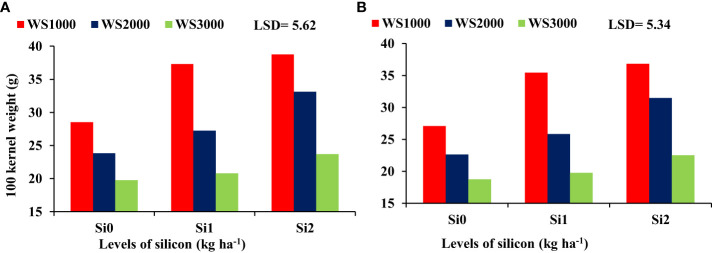
Effect of silicon on the 100 kernel weight under salt stress during 2019-20 **(A)** and 2020-21 **(B)**.

**Figure 9:**
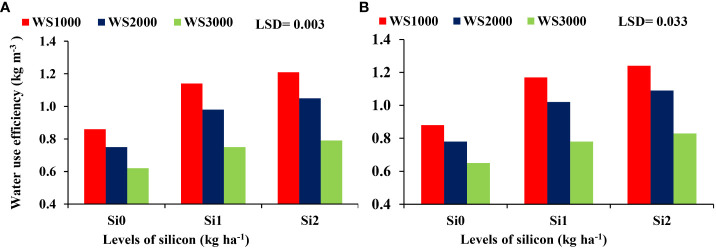
Effect silicon on the WUE under salt stress during 2019-20 **(A)** and 2020-21 **(B)**.

There were no significant differences regarding the interaction effect between WS and Si on the HI, and shelling percentage, whereas 100-kernel weight (**Figure 8**) and WUE (**Figure 9**) were significant. The mean values of WUE and 100-kernel weight increased with an increase in Si concentration under all salinity levels. Si application at all concentrations of 0, 1, and 2 kg ha^-1^ increased the 100-kernel weight, and the maximum WUE of maize was observed under a salinity level of 1000 mg L^-1^, and the minimum values were under a salinity level of 3000 mg L^-1^. However, the highest WUE was recorded with the highest concentration of Si (2 kg ha^-1^) under all salinity levels.

## Discussion

In this study, irrigation with saline water had negative effects on all the components of growth and yield. The severe impacts of irrigation water salinity on theyield components are compatible with Schubert et al. (2009) who found that, salinity resulted in poor kernel setting with reduced grain number, weight, and low grain yield of maize. Irrigation of maize plants with saline water resulted in significant reduction in cob length, cob diameter, number of seeds per cob and 100 seed weight at high salinity levels (Ashrafuzzaman and Khan, 2000). Reduction in maize yield by salinity is attributed to many factors; it may be due to osmotic problems, or due to difficulty in plant absorption of water from the soil (Schubert et al. (2009). Also, Hussain et al. (2019) reported that, plant growth under salinity stress is affected in three ways, the osmotic stress and increase of phytotoxic ions, the ionic stress in the cytosol, and the oxidative stress by reactive oxygen species (ROS), and these factors cause a reduction in plant water uptake, in ion and hormone imbalance, reduction of photosynthesis, and finally reduction of the plant growth and yield. Furthermore, an imbalance in Na and K ions uptake by plants under salinity stress particularly when reaching high levels causes many physiological problems in plant roots, leaves, grains, and fruits (James et al., 2011).

It was noted that results in this study had similarities with Amin et al. (2018), in that Si application resulted in significant increases in cob fresh and dry weight, and total fresh and dry weight. In addition, there was a significant highest green and dry shoot yield of maize plants with the application of Si at a rate of 300 mg Si kg^-1^ soil (Meena et al., 2014). Similarly, Janislampi (2012) also recorded significant increase in maize dry mass by up to 18% after the application of Si as findings. This was due to the deposition of Si in plant leaves and reduction of the transpiration rate due to stomata closure, dilution of the salts accumulated in the saline environment, and an increase in crop production (Ali et al., 2012).

The results in this study indicate that the foliar application of Si contributed significantly in alleviating salinity stress on maize biomass (Fresh and dry weight of cob and total biomass) which is consistent with findings by Raza et al. (2019), who found Si application increased maize plant growth parameters that were otherwise reduced under saline conditions. Sattar et al. (2020), found that applying and spraying Si on the leaves alleviated salinity stress in wheat and mungbean plants. Ahmad et al. (2013), found significant increases after applying Si in the chlorophyll content, leaf area index, root dry weight, leaf dry weight, shoot dry weight, total dry weight, and specific leaf weight, compared with control. Whereas, Ahmed et al. (2011), ascribed that the important role of Si application as regards plant growth is that it enhances and improves water uptake by plants, and by this means plants can withstand salinity and drought stress. Shi et al. (2014) added that Si enhances and balances plant uptake of nutrients from the soil under salinity and drought stress. Also, Sonobe et al. (2017) found that the treatment of sorghum plants growing under salinity stress with Si absorbed more water due to reduced osmotic potential in the roots compared with the control, and this resulted in increased dry weight of the Si treated plants.

The results obtained in this research work emphasizing the positive and alleviating effects of foliar application of Si on yield components of maize grown under salinity effects agree with the findings by Kaya et al. (2006) who found that Si has enhanced the morphological characteristics and net yield.

Mean values of water use efficiency (WUE) were significantly decreased with an increase in WS levels. Similar results were also found by Amer and El-Emary (2018), who reported that the WUE of maize was decreased due to increasing irrigation water salinity. Si application in this study resulted in significant increases in WUE which agreed with the results obtained by Janislampi (2012), who reported that Si increased WUE in maize by up to 36%. Also, Gao et al. (2005) reported that the influence of Si on WUE in maize plants was investigated and the results showed that plants treated with 2 mmol L^-1^ Si had 20% higher WUE than that plants without Si application. The WUE of maize increased by foliar Si application by 17.65 and 18.75 compared to the control treatment in the first and second seasons, respectively (Amer and El-Emary, 2018). These increases in WUE may be due to the positive effects of Si application on increasing the grain yield of maize and reducing the adverse effects of irrigation water salinity on the growth and yield of maize according to Roohizadeh et al. (2015). Furthermore, this improvement is because Si improved the performance of defense mechanisms in maize plants under salinity stress and this led to the alleviation of both osmotic and oxidative stress in maize crops (Khan et al., 2018).

## Conclusions

In the present study, irrigation with saline water had negative effects on WUE and resulted in a significant decrease in all components of growth and yield in all maize cultivars. In contrast, the application of foliar Si to the salt-stressed maize cultivars significantly attenuated the harsh and adverse effects of salinity and increased the WUE, plant growth, yield components, and yield of maize cultivars. From this study, it is concluded that foliar application of Si (2 kg Si ha^-1^) alleviates the detrminal impacts of irrigation water salinity on the growth and yield parameters of maize under natual field conditions.

## Data availability statement

The datasets presented in this study can be found in online repositories. The names of the repository/repositories and accession number(s) can be found in the article/supplementary material.

## Author contributions

All authors listed have made a substantial, direct, and intellectual contribution to the work and approved it for publication.

## Funding

Institutional Fund Projects under grant no. (IFPIP- 690-155-1443) by the Ministry of Education and King Abdulaziz University, DSR, Jeddah, Saudi Arabia.

## Acknowledgments

This research work was funded by Institutional Fund Projects under grant no. (IFPIP: 690-155-1443). The authors gratefully acknowledge technical and financial support provided by the Ministry of Education and King Abdulaziz University, DSR, Jeddah, Saudi Arabia.

## Conflict of interest

The authors declare that the research was conducted in the absence of any commercial or financial relationships that could be construed as a potential conflict of interest.

## Publisher’s note

All claims expressed in this article are solely those of the authors and do not necessarily represent those of their affiliated organizations, or those of the publisher, the editors and the reviewers. Any product that may be evaluated in this article, or claim that may be made by its manufacturer, is not guaranteed or endorsed by the publisher.
